# Prevalence and Antibiotic Resistance of Klebsiella pneumoniae in Diabetic Foot Ulcer

**DOI:** 10.7759/cureus.67824

**Published:** 2024-08-26

**Authors:** Shivani Reddy P, Carmelin Durai Singh, Sathish Kumar, Raman Muthusamy

**Affiliations:** 1 Center for Global Health Research, Saveetha Medical College and Hospital, Saveetha Institute of Medical and Technical Sciences, Chennai, IND; 2 Neurology, Saveetha Medical College and Hospital, Saveetha Institute of Medical and Technical Sciences, Chennai, IND

**Keywords:** carbapenam, bacterial virulence, diabetic foot ulcers (dfus), klebsiella pneumoniae (kp), antimicrobial resistance

## Abstract

Introduction

A serious global threat of antimicrobial resistance has emerged due to the improper use of antibiotics, including polypharmacy and inappropriate prescribing. This misuse has led bacteria to develop immunity against these drugs. *Klebsiella pneumoniae*, a concerning gram-negative bacterium, has become resistant, especially among immunocompromised diabetic patients for multiple antibiotics. To fight effectively this growing crisis and regain control of these infections, it is crucial to comprehend the resistance mechanisms utilized by the bacteria and develop a new therapeutic strategy to prevent antibiotic resistance.

Materials and methods

A five-month study from January 2023 to May 2023 was conducted at the tertiary healthcare facility of Saveetha Medical College by collecting 122 clinical specimens from patients with diabetic foot ulcers (DFUs) and ulcer-related infections. The microbiological testing methods followed by the identification of bacteria using matrix-assisted laser desorption/ionization time-of-flight mass spectrometry (MALDI-TOF) and antimicrobial susceptibility testing (AST) by the VITEK 2 Compact system were performed.

Results

A stout, rod-shaped, gram-negative bacilli was observed in gram staining, and growth of mucoid γ-hemolytic colonies and lactose-fermenting mucoid colonies were seen in blood and MacConkey agar plates. MALDI-TOF analysis confirmed the presence of *Klebsiella pneumoniae* along with other bacteria such as *Klebsiella oxytoca*, *Staphylococcus* spp., *Proteus* spp., and *​​Escherichia ​coli*. VITEK showed high resistance rates to commonly used antibiotics, including carbapenems. Notably, isolates showed sensitivity and intermediate to tigecycline and colistin. Resistance patterns varied across specimen types, emphasizing the importance of considering clinical sources when interpreting data.

Conclusion

Hence, this study underscores the urgent need for novel antimicrobial agents and effective infection control measures to combat multidrug-resistant *Klebsiella pneumoniae* infections. Understanding resistance mechanisms is essential for the incorporation of treatment strategies and preserving antibiotic efficacy.

## Introduction

The occurrence of diabetic foot ulcers (DFUs) is strongly linked to complications in the lower extremities, particularly diabetic polyneuropathy (DPN) and diabetic peripheral artery disease (DPAD) [1]. The primary reason for DFUs was due to prolonged hospitalization, which was observed globally, especially with a greater impact in developing countries such as India [2]. According to research, it further highlights the concerning rise in hospitalization rates due to diabetic foot infections, emphasizing the need for improved preventive measures and early intervention strategies [3]. These ulcers occur mostly as open sores on the bottom of the feet. These wounds are caused by the complex interplay of peripheral neuropathy and peripheral arterial disease, which are the common complications of diabetes [4]. Diabetic neuropathy foot ulcers mainly affect the sensory, motor, and sudomotor functioning of the feet. As a consequence of this dry skin, decreased pain perception and altered biomechanics of the foot lead to ulcer formation [5]. Dry skin, cracks, and fissures occur mainly due to the involvement of autonomic fibers that cause anhidrosis [6]. The triad of diabetic neuropathy, peripheral arterial ischemia, and secondary infection also contribute to foot ulceration [7].

The other serious complication of DFU is due to the infection caused by polymicrobial pathogens, especially, aerobic gram-negative bacilli such as *Klebsiella pneumoniae*, *Escherichia coli, *and *Pseudomonas aeruginosa* and also gram-positive cocci *Staphylococcus aureus* [8,9]. Bacterial pathogens have the ability to survive within the wound by forming an extracellular self-protective layer of microbial communities. *Klebsiella pneumoniae*, an opportunistic pathogen, develops a thick layer of biofilm, which is one of the virulent factors that contribute to drug resistance [10]. These multidrug-resistant or extensively drug-resistant pathogens are defined by the term "superbugs," because they exhibit resistance to a broad spectrum of antibiotics, which renders many current treatments ineffective [11]. In low- and middle-income countries, for example, the misuse of antibiotics due to factors such as over-the-counter sales and lack of regulation has led to higher rates of resistant infections. In South Asian and African regions, studies have shown a distributing rise in drug-resistant organisms in diabetic foot infections (DFIs), which complicates treatment and increases the risk of severe complications, including amputations. Cultural practices, such as the use of traditional medicine and the delay in seeking professional healthcare, further contribute to the spread of resistant infection areas [3]. It is an unavoidable evolutionary phenomenon, where microbes develop genetic mutations to survive against antimicrobial agents. The overuse and misuse of antimicrobials, especially antibiotics, have provided ample opportunity for bacteria to develop resistance. As a consequence, there is an increase in both incidence and prevalence of infections caused by bacteria resistant to these drugs. These antimicrobial-resistant infections pose a significant challenge, often requiring more complex treatment strategies and carrying a higher risk of complications, even death [12]. In addition to this, inadequate microbiological information drives the use of broad-spectrum antibiotics, fostering the emergence of resistant strains [13].

Enzymatic antibiotic activation, porin loss, efflux pump overexpression, carbapenem resistance genes, and extended-spectrum beta-lactamase (ESBL) genes that make *Klebsiella pneumoniae* resistant to cephalosporins and monobactams limit treatment options [14-16]. These mechanisms and genes collectively contribute to the antibiotic resistance profile of *Klebsiella pneumoniae*, making it a challenging pathogen to treat effectively with conventional antibiotics. Understanding these mechanisms is crucial for developing targeted prevention strategies and novel control measures against this pathogen.

## Materials and methods

For bacterial isolation and confirmation, blood agar, MacConkey agar, and selective biochemical reagents were purchased from Hi-Media (Mumbai, India). Standard isolated* Klebsiella pneumoniae* was obtained from the Department of Microbiology, Saveetha Medical College. For bacterial identification and to determine antibiotic resistance, matrix-assisted laser desorption/ionization time-of-flight mass spectrometry (MALDI-TOF) was performed at the Gujarat Biotechnology and Scientific Research Centre, Gandhinagar, Gujarat, and VITEK was performed at the Saveetha Medical College and Hospital Laboratory Complex. Statistical analysis was performed for the obtained samples.

Collection of samples

A total of 122 samples, which included biopsies, were collected during the specialized diagnostic sessions. Wound swabs from the foot ulcers and blood samples were collected from DFU patients suspected of infections due to *Klebsiella* spp. during the evaluation conducted on routine visits who attended the tertiary healthcare setting of Saveetha Medical College and Hospital (SMCH). The necessary approval was obtained from the Institutional Review Board for carrying out this study program (IRB number: 112101159/SMC/SIMATS).

Materials and methods

Patients presented with ulceration complaints, such as DFU, and ulcers due to immobilization and other ulcer-related conditions were examined. Over a five-month period from January 2023 to May 2023, the specimens were collected from these patients at the physician's discretion, and their microbiological data was analyzed. The culture reports and antimicrobial susceptibility patterns of all *Klebsiella* isolates were evaluated. Bacterial colonies were identified using matrix-assisted laser desorption/ionization time-of-flight mass spectrometry (MALDI-TOF), and antimicrobial susceptibility testing (AST) was performed using the VITEK system. The antimicrobial tested included amoxicillin/clavulanic acid, piperacillin-tazobactam, cefuroxime, cefuroxime axetil, ceftriaxone, cefoperazone/sulbactam, cefepime, ertapenem, imipenem, meropenem, amikacin, gentamicin, minocycline, ciprofloxacin, tigecycline, colistin, and trimethoprim-sulfamethoxazole.

Inclusion criteria

Patients who gave consent to participate were included in the study. This study included patients at the age of 18 years and above, with confirmed diagnosis of diabetes mellitus, and with clinically diagnosed DFU. Samples were collected as wound swabs, biopsies, and blood for microbiological analysis.

Exclusion criteria

Patients without a confirmed diagnosis of diabetes mellitus; patients who have received systemic antibiotics within the last two weeks prior to sample collection to avoid influence on microbial cultures and resistance patterns; patients with conditions that severely compromise the immune system, such as HIV/AIDS or active cancer treatment; patients with severe commodities that may independently affect infection rates or antibiotic resistance patterns, such as advanced chronic kidney diseases or hepatic failure; and pregnant women were excluded from the study.

Sample size calculation

Based on the study design, we have selected a sample size of 122 diabetic foot ulcer patients to determine the prevalence and antibiotic resistance of *Klebsiella pneumoniae*. Using a 95% confidence level and assuming an estimated prevalence of 50%, the sample size provides a margin of error of approximately ±8.87%. This margin of error is considered acceptable for the study's objectives.

## Results

**Figure 1:**
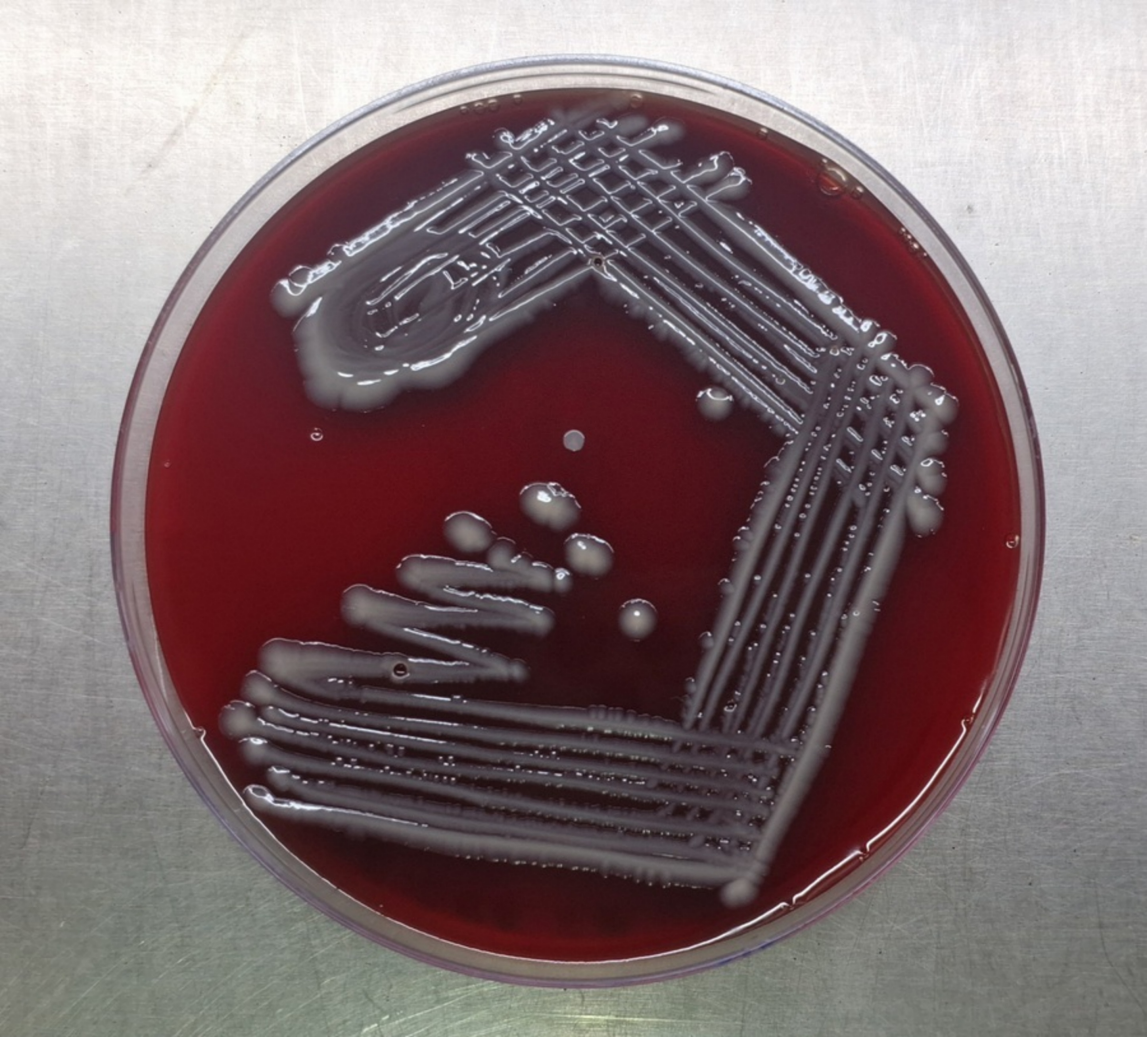
Klebsiella pneumoniae isolate on blood agar plate

**Figure 2:**
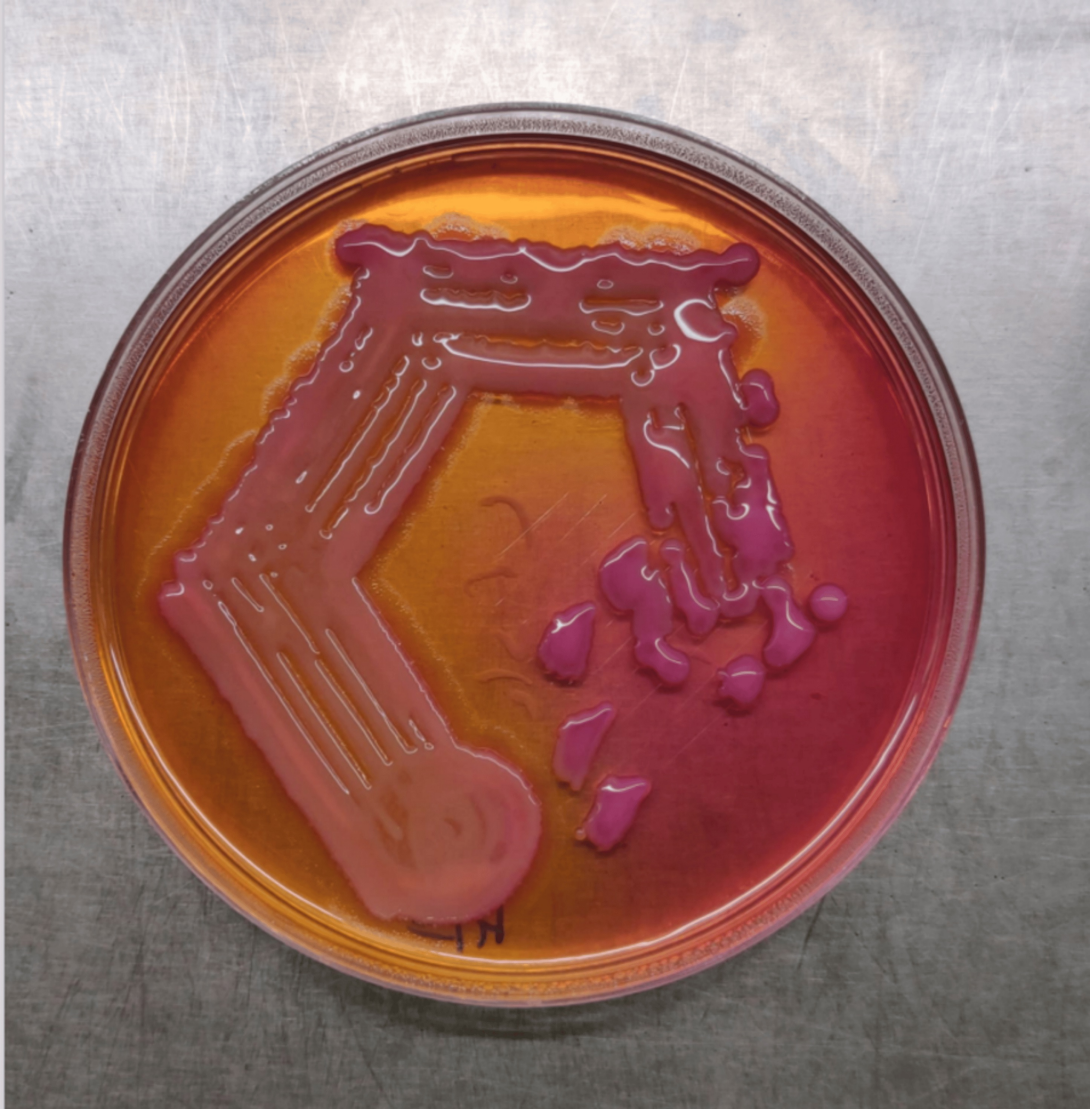
Klebsiella pneumoniae isolate on MaCconkey agar plate

After the microbiological testing assays were performed, the presence of stout, rod-shaped, gram-negative bacilli of *Klebsiella* spp. was observed. Mucoid γ-hemolytic colonies (Figure 1) and lactose-fermenting mucoid colonies (Figure 2) were identified in blood agar and MacConkey agar plates, respectively.

Among the cultured samples, *Klebsiella pneumoniae* was found in wound swabs (n=40), biopsy (n=24), and blood (n=13). Along with this, *Escherichia ​coli* (n=8) in blood, *Proteus* spp. (n=10) in biopsy, and *Staphylococcus* spp. (n=19) in wound swabs were also identified as depicted in Table 1.

**Table 1:** Total number of samples collected from various sites

Type of sample	Number of samples	Positive organisms
Wound swabs	40	Klebsiella pneumoniae
Biopsy	24	Klebsiella pneumoniae
Blood	13	Klebsiella pneumoniae
Biopsy	8	Klebsiella oxytoca
Blood	8	Escherichia coli
Biopsy	10	*Proteus* spp.
Wound swabs	19	*Staphylococcus* spp.

The isolated colonies were further confirmed as *Klebsiella pneumoniae* by MALDI-TOF, along with the other species such as* Klebsiella oxytoca*, *Staphylococcus* spp., *Proteus* spp., and *E. coli*,* *as represented in Table 2.

**Table 2:** Bacterial identification by MALDI-TOF MALDI-TOF: matrix-assisted laser desorption/ionization time-of-flight mass spectrometry, ST: strain

Strain number	Results of MALDI-TOF
ST 01-ST08	99.8% *Staphylococcus* spp.
ST 09-ST44	99.7% *Klebsiella pneumoniae*
ST 45-ST47	95.9% *Klebsiella oxytoca*
ST 48-ST 53	92.7% *Proteus* spp.
ST 54-ST 64	93% *Staphylococcus* spp.
ST 65-ST 69	99.2% *Klebsiella oxytoca*
ST 70-ST 71	92.8% *Escherichia coli*
ST 72-ST 79	93.4% *Klebsiella pneumoniae*
ST 80-ST 82	99.7% *Escherichia coli*
ST 83-ST 92	91.3% *Klebsiella pneumoniae*
ST 93-ST 105	93.2% *Klebsiella pneumoniae*
ST 106-ST 109	92.4% *Proteus* spp.
ST 110-ST 114	93.7% *Klebsiella pneumoniae*
ST 115-ST 117	92.6% *Escherichia coli*
ST118-ST 122	93.2% *Klebsiella pneumoniae*

To assess the antibiotic resistance profile of the* Klebsiella pneumoniae* isolates, VITEK was performed against a panel of antimicrobial agents, which includes amoxicillin/clavulanic acid, piperacillin-tazobactam, cefuroxime, cefuroxime axetil, ceftriaxone, cefoperazone/sulbactam, cefepime, ertapenem, imipenem, meropenem, amikacin, gentamicin, minocycline, ciprofloxacin, tigecycline, colistin, and trimethoprim-sulfamethoxazole. The results of the antibiotic susceptibility testing revealed a concerning pattern of multidrug resistance among the *Klebsiella pneumoniae* isolates. A high percentage of the isolates demonstrated resistance to commonly used antibiotics, such as amoxicillin/clavulanic acid, piperacillin-tazobactam, cefuroxime, cefuroxime axetil, ceftriaxone, amikacin, gentamicin, ciprofloxacin, and trimethoprim-sulfamethoxazole. Additionally, a significant proportion of the isolates exhibited resistance to the carbapenems group including ertapenem, imipenem, and meropenem, which are often considered the last line of defense against gram-negative bacterial infections. Also, sensitivity was observed for tigecycline and intermediates in colistin. The antibiotic resistance pattern is represented in Table 3.

**Table 3:** Antibiotic resistance of Klebsiella pneumoniae-VITEK MIC: minimum inhibitory concentration, R: resistant, S: sensitive, I: intermediate

Antimicrobial	MIC	Interpretations
Amoxicillin/clavulanic acid	>=32	R
Piperacillin-tazobactam	>=128	R
Cefuroxime	>=64	R
Cefuroxime axetil	>=64	R
Ceftriaxone	>=64	R
Cefoperazone/sulbactam	>=64	R
Cefepime	>=32	R
Ertapenem	>=8	R
Imipenem	8	R
Meropenem	>=16	R
Amikacin	32	R
Gentamicin	>=16	R
Ciprofloxacin	>=4	R
Tigecycline	2	S
Colistin	2	I
Trimethoprim-sulfamethoxazole	>=320	R

The study also highlighted the variable resistance patterns observed in isolates from wound ulcers, exhibiting higher resistance rates compared to those from other sources, such as blood. These findings underscore the importance of considering the clinical source of the isolates when interpreting antibiotic-resistant data and guiding appropriate treatment strategies. The comprehensive assessment of the antibiotic resistance of *Klebsiella pneumoniae* isolates in this study provides valuable insights into the evolving antimicrobial resistance of this critical pathogen. These findings emphasize the urgent need for the development of novel antimicrobial agents, as well as the implications of effective infection control measures and antimicrobial stewardship programs to combat the growing threat of multidrug-resistant *Klebsiella pneumoniae* infections.

Statistical analysis

This analysis of standard deviation provides the accuracy and variability of the MALDI-TOF results across differential bacterial strains identified in the samples. *Klebsiella pneumoniae* showed a wide range of identification percentages with a minimum of 91.3% and a maximum of 99.7%. The most common infections of *E. coli *and *Staphylococcus* spp. had the highest maximum identification percentages of 99.7% and 99.8% respectively. *Proteus* spp. had the lowest variability in identification, as shown by the standard deviation of 0.21. *Klebsiella oxytoca* had the highest mean identification rate at 97.55%, with a relatively low standard deviation, indicating consistent identification accuracy as depicted in Table 4. However, the study concentrated on the multidrug resistance of *Klebsiella pneumoniae, *which showed a wide range of strains.

**Table 4:** Statistical analysis of identified strain (standard deviation method)

Strain identification	Number	Mean (%)	Standard deviation	Minimum (%)	Maximum (%)
Escherichia coli	3	95.03	4.04	92.6	99.7
Klebsiella oxytoca	2	97.55	2.33	95.9	99.2
Klebsiella pneumoniae	6	94.08	2.88	91.3	99.7
*Proteus* spp.	2	92.55	0.21	92.4	92.7
*Staphylococcus* spp.	2	92.55	4.81	93.0	99.8

## Discussion

*Klebsiella pneumoniae* is an eminent nosocomial, gram-negative bacterial pathogen responsible for a diverse array of infections. Its escalating resistance to antimicrobials has emerged as a significant public health threat [17]. This study aimed to characterize 122 clinical *Klebsiella pneumoniae* isolates obtained from the various types of samples of DFU patients. Based on their susceptibility, a comprehensive panel of antibiotics and the presence of various virulence factors associated with the pathogenesis should be investigated [18]. The findings from this comparative analysis will enhance our understanding of the antimicrobial resistance and virulence potential of *Klebsiella pneumoniae* strains associated with DFU. As per the previous study conducted, multiantibiotic-resistant bacteria are mostly found in the wound site of DFU patients [19]. Similarly, this study shows more resistance to the strains obtained from the wound swab samples.

*Klebsiella pneumoniae* shows the existence of two distinct types of pathogen, classical type (cKp) and hypervirulent (hvKp), strains in which *rmpA* and *iucA* genes serve as biomarkers for their differentiation [20]. The new *Klebsiella pneumoniae* strains combine the aggressive nature of hypervirulent *K. pneumoniae* (hvKP) with the multidrug-resistant cKp. These hybrid strains pose a serious threat as they can cause severe, widespread infections in healthy people and are resistant to many antibiotics, making treatment challenging. Researchers have confirmed the existence of hvKP strains that have acquired genes responsible for extended-spectrum beta-lactamase (ESBL) or carbapenemase production, highlighting the concerning potential of these convergent strains [21-24]. It reflects a fundamental difference in how these bacteria interact with the host and cause diseases [25]. The core of the pathogen distinction lies in their virulence factors, the specialized molecules produced by* Klebsiella pneumoniae* immune evasion, and tissue damage. cKp and hvKp possess distinct virulence factors, leading to contrasting phenotypes and clinical presentations. The virulent factors of cKp strains typically rely on a border set of virulence factors, including capsular polysaccharides (K antigens), lipopolysaccharides (LPS), fimbriae, and iron acquisition systems (siderophores) [26]. These factors primarily facilitate adherence to host tissue, resist phagocytosis by immune cells, and acquire essential nutrition for bacterial growth. cKp are more commonly associated with hospital-acquired (nosocomial) settings and tend to affect immunocompromised individuals. hvKp strains are characterized by a more streamlined virulence arsenal, often featuring a hypermucoviscous capsule due to the presence of the *rmpA* gene and siderophore aerobactin [27]. This potent capsule provides exceptional resistance to phagocytosis and immune defenses. Additionally, hvKp may possess toxins such as cytotoxic necrotizing factor (CNF-1), which directly damage host tissues. These infections can involve the lungs (pneumonia), bloodstream (bacteremia), liver (abscesses), and nervous system (meningitis) [28].

Researchers have found a high prevalence of resistance to commonly used antibiotics such as ampicillin, ceftazidime, and ciprofloxacin. The majority of antimicrobial resistance of *Klebsiella pneumoniae *in DFU patients was found to be with amoxicillin/clavulanic acid, piperacillin-tazobactam, cefuroxime, cefuroxime axetil, ceftriaxone, amikacin, gentamicin, ciprofloxacin, and trimethoprim-sulfamethoxazole [29]. Currently, the prevalence of ESBL-producing *Klebsiella pneumoniae* is a growing concern worldwide. These antibiotic-resistant bacteria are becoming increasingly common, with some regions reporting staggering rates exceeding 50% of *Klebsiella pneumoniae* isolates exhibiting the resistance mechanism [30]. However, this study showed that the infection of DFU by *Klebsiella pneumoniae* also reflects some level of resistance to carbapenems. This highlights the urgent need for effective treatment strategies and improved antibiotic stewardship practices to combat this global threat.

Limitations

The limited number of samples, which restricts the study findings, may not be generalizable to the entire population of DFU patients at the healthcare facility or to a broader diabetic population. A larger sample size would provide a more accurate representation of the prevalence and resistance patterns. Focusing on a specific set of virulence factors associated with *Klebsiella* pathogenesis, a more comprehensive analysis of virulence factors could provide deeper insights into the strain's pathogenic potential. The study being conducted at a single tertiary healthcare facility limits the generalizability of the findings to other healthcare settings or geographical regions. Future research will include multicenter studies to enhance generalizability, by further exploring specific virulence factors of *Klebsiella pneumoniae* through genotypic studies and evaluating the alternative treatments, and conduct longitudinal studies to improve understanding and management of infections in diverse settings.

## Conclusions

This study characterized* Klebsiella pneumoniae* isolates from DFU and evaluated their antimicrobial resistance and their virulence factors. Our findings confirm a high prevalence of resistance to commonly used antibiotics, including amoxicillin/clavulanic acid, piperacillin-tazobactam, cefuroxime, cefuroxime axetil, ceftriaxone, amikacin, gentamicin, ciprofloxacin, and trimethoprim-sulfamethoxazole. Notably, a concerning level of resistance to the last resort of antibiotics, carbapenems, was also observed. These findings align with previous studies highlighting the growing challenge of antibiotic-resistant *Klebsiella pneumoniae* in DFU infections. The emergence of carbapenem resistance further emphasizes the critical need for enhanced surveillance of antimicrobial resistance patterns in *Klebsiella pneumoniae* isolated from DFUs. Additionally, developing alternative treatment strategies for these complex infections becomes crucial. Therefore, improved infection control measures, antimicrobial stewardship programs, and the development of novel antibiotics should be implemented.
